# Two-photon polymerization for 3D biomedical scaffolds: Overview and updates

**DOI:** 10.3389/fbioe.2022.994355

**Published:** 2022-08-22

**Authors:** Xian Jing, Hongxun Fu, Baojun Yu, Meiyan Sun, Liye Wang

**Affiliations:** ^1^ Key Laboratory of Micro/Nano and Ultra-precision Manufacturing, School of Mechatronic Engineering, Changchun University of Technology, Changchun, Jilin, China; ^2^ College of Laboratory Medicine, Jilin Medical University, Jilin, China; ^3^ College of Pharmacy, University of Houston, Houston, TX, United States

**Keywords:** two-photon, photopolymerization, microfabrication, scaffolder, multidisciplinary field

## Abstract

The needs for high-resolution, well-defined and complex 3D microstructures in diverse fields call for the rapid development of novel 3D microfabrication techniques. Among those, two-photon polymerization (TPP) attracted extensive attention owing to its unique and useful characteristics. As an approach to implementing additive manufacturing, TPP has truly 3D writing ability to fabricate artificially designed constructs with arbitrary geometry. The spatial resolution of the manufactured structures *via* TPP can exceed the diffraction limit. The 3D structures fabricated by TPP could properly mimic the microenvironment of natural extracellular matrix, providing powerful tools for the study of cell behavior. TPP can meet the requirements of manufacturing technique for 3D scaffolds (engineering cell culture matrices) used in cytobiology, tissue engineering and regenerative medicine. In this review, we demonstrated the development in 3D microfabrication techniques and we presented an overview of the applications of TPP as an advanced manufacturing technique in complex 3D biomedical scaffolds fabrication. Given this multidisciplinary field, we discussed the perspectives of physics, materials science, chemistry, biomedicine and mechanical engineering. Additionally, we dived into the principles of tow-photon absorption (TPA) and TPP, requirements of 3D biomedical scaffolders, developed-to-date materials and chemical approaches used by TPP and manufacturing strategies based on mechanical engineering. In the end, we draw out the limitations of TPP on 3D manufacturing for now along with some prospects of its future outlook towards the fabrication of 3D biomedical scaffolds.

## Introduction

For more than half a century, the needs for high-resolution, well-defined and complex three-dimensional (3D) microstructures in diverse fields such as information technology, electronics, photonics, and micro-electromechanical systems (MEMS), bionics and biomedical microdevices have led to the rapid development of many novel 3D microfabrication techniques (LaFratta et al., 2007; Lee K.-S. et al., 2008; Dvurechenskii and Yakimov, 2017; Fritzler and Prinz, 2019).

When mentioning micro/nanomanufacturing, photolithography has been thought as the dominant technology in microfabrication for a long time. By shrinking the size of the products, photolithography makes a leap forward in the manufacture of electronic components. However, both the traditional photolithography and other lithography techniques derived from or substituted for it, including dip-pen nanolithography (Ginger et al., 2004), capillary force lithography (Kim et al., 2001), nanoimprint lithography (Dvurechenskii and Yakimov, 2017; Schift, 2008), soft lithography (Geissler and Xia, 2004), transfer lithography (Yao et al., 2013; Yao et al., 2021) and others, are inherently two-dimensional. Features currently available in 3D structures using these methods have not be comparable to what can be achieved in 2D (LaFratta et al., 2007).

Other important approaches developed for 3D fabrication/microfabrication include the LIGA process (Singleton, 2003), self-assembly (Xia et al., 2000; Wang et al., 2002), holographic lithography (Shoji and Kawata, 2000; Shoji et al., 2003) phase-mask lithography (Jeon et al., 2004), and a relatively broad category of techniques, three-dimensional printing (3DP) (Schubert et al., 2014; Gibson et al., 2015; Laird et al., 2021; Yang et al., 2021). Among these important methods mentioned above, the LIGA process is a combination of lithography, electroplating, and molding, which possesses the ability to fabricate simple 3D structures with smooth surfaces and sharp vertical features, however, it is incapable of making complex structures. Self-assembly is an autonomous organization of components into 3D structures that have Micro/nano characteristics without human intervention. Although numerous studies have demonstrated its feasibility, some difficulties remain in obtaining specific aperiodic constructions from complex and diverse self-assembly systems. Both holographic lithography and phase-mask lithography take advantage of laser interference to produce periodic patterns in photoresist to create periodic structures, but the arbitrary ones cannot be formed in 3D space.

3DP, invented back in the 1980s by Charles Hull (Hull, 1986), has been developed into three branches: inkjet-, syringe- and light-based 3DP (Jonušauskas et al., 2018; Van Hoorick et al., 2019). 3DP refers to multiple specific methods, the most common used of which are inkjet printing (IJP) (Derby, 2010; Gibson et al., 2015), fused deposition modeling (FDM) (Wong and Hernandez, 2012; Masood, 2014), selective laser sintering (SLS) (Wong and Hernandez, 2012), (micro)stereolithography (SLA) (Wong and Hernandez, 2012; Zhou et al., 2016), and digital light processing (DLP) (Hornbeck et al., 1997; Houben et al., 2017). These techniques are often described as lay-by-lay techniques, because of the formation of 3D structures involving sequential horizontal layering of a series of layers (or slices) on top of one another (Laird et al., 2021). As a consequence, the two-dimensional nature of fabrication inevitably imposes geometric limitations on 3D structures made using these techniques (LaFratta et al., 2007). In addition, although these techniques can produce fully 3D structures, it’s hard to provide a better resolution over a few microns (Baldacchini et al., 2021).

Due to the limitations of these traditional 3D fabrication techniques, two-photon polymerization (TPP) has attracted extensive attention owing to its unique and useful characteristics since it was developed in 1997 (Maruo et al., 1997a; Baldacchini et al., 2021). Over the past 2 decades, TPP has been evolved to a practical method widely employed in many fields (Baldacchini, 2015; Stampfl et al., 2016) and a great deal of results have been obtained such as photonic crystals, microfluidic components, micro-and nanorobots and biomedical scaffolds (Ovsianikov et al., 2011a; Soukoulis and Wegener, 2011; Wang et al., 2018; Sala et al., 2021). TPP is based on the optical nonlinear absorption (usually a near-infrared femtosecond laser) to induce polymerization or crosslinking in the photopolymerizable materials. When a femtosecond laser is tightly focused into the material, one photoinitiator (PI) molecule simultaneously absorbs two photons to be excited to initiate local free-radical polymerization within the focus volume, it breaks free from the lay-by-lay paradigm and achieves truly 3D arbitrary structure writing (Van Hoorick et al., 2019; Baldacchini et al., 2021). Taking advantage of the two-photon absorption (TPA) probability which is proportional to the square of the intensity and threshold character of the polymerization process, TPP can process constructs with spatial resolution less than the diffraction limit (Fischer and Wegener, 2013; Fritzler and Prinz, 2019; Baldacchini et al., 2021). The smallest resolution that is relatively easy to achieve using TPP is about several hundred nanometers and sub-100 nm resolution has been repeatedly demonstrated (Kawata et al., 2001; Li et al., 2009; Sakellari et al., 2012). Because of these characteristics, TPP is outstanding for the fabrication of 3D biomedical structures and plays a pivotal role in the engineering cell culture matrices which are needed to properly mimic the microenvironments of natural extracellular matrix (ECM) in order to investigate a plethora of biomedical problems (Ovsianikov et al., 2012). Other traditional techniques for producing 3D biomedical structures, including phase separation, particulate leaching, solvent casting and freeze-drying, have relatively low spatial resolution compared with TPP, generally tens of microns (cellular scale) (Song et al., 2021). Electrospinning can be used to manufacture fibers on nano scale and this technique has been widely used in the fabrication of 3D biomedical scaffolds (Zou et al., 2021; Zou et al., 2022). However, the dense accumulation of fibers will hinder the cellularization process (Weisgrab et al., 2020). At the same time, these traditional techniques have considerable difficulties in accurately reproducing complex 3D computer-aided design (CAD) models and precisely defining the geometry of biomedical scaffolds. At a subcellular scale (1–10 μm) and in well-defined 3D structures to investigate cell behavior (proliferation, differentiation, migration, and adhesion) responding to the physicochemical and biological characteristics of their surrounding environment has been a broad consensus in the biomedical field (Ovsianikov et al., 2012; Qin et al., 2014b; Akhmanova et al., 2015).

In this review, we presented an overview of the progress and application of TPP as an advanced manufacturing technique in complex 3D biomedical scaffolds fabrication. Specifically, after introducing the mechanisms of TPA and TPP, we draw our attention to developed-to-date materials and chemical approaches for the fabrication of 3D biomedical scaffolds using TPP. Subsequently, we described the typical 3D architectures produced of varieties of TPP processable materials for applications in cytobiology, tissue engineering and regenerative medicine, focusing on the various manufacturing strategies based on mechanical engineering used in the processing.

## Two-photon absorption and two-photon polymerization

**FIGURE 1:**
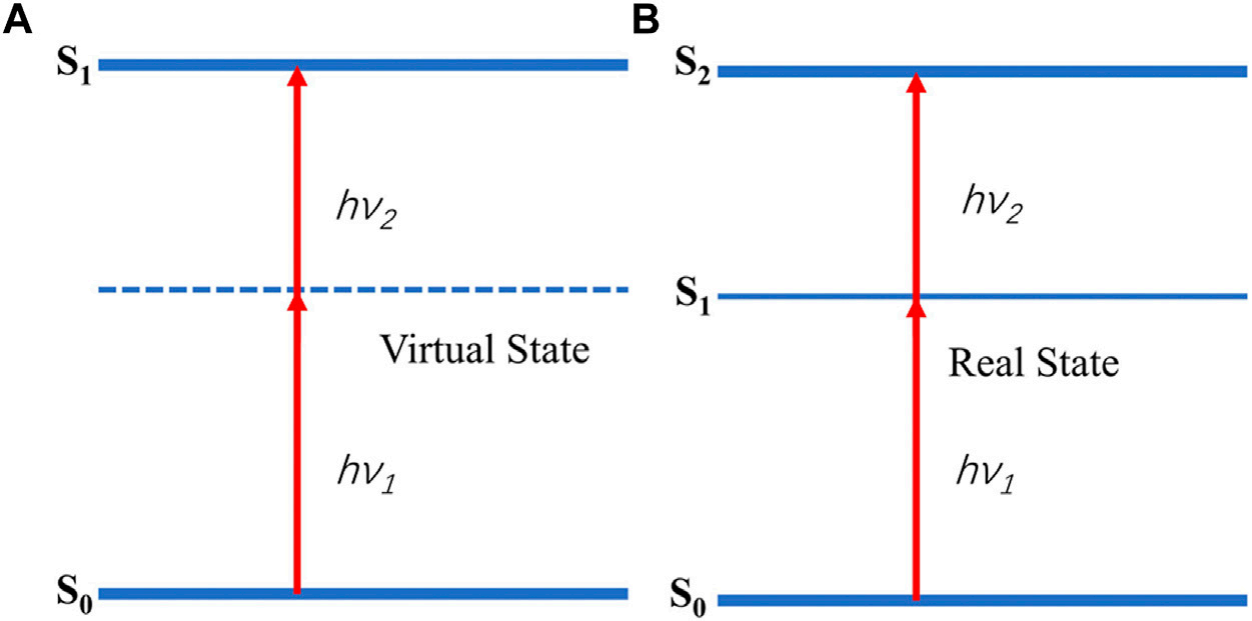
Simultaneous **(A)** and sequential absorption. **(B)**. hν1 = hν2:degenerate case; hν1≠hν2:nondegenerate case.

In fact, TPP can be carried out through multiple mechanisms, TPA is just one of them (Nguyen and Narayan, 2017). This paper is intended to review the processing technique of complex 3D scaffolds relying on the TPA in the photopolymerizable materials, so the following descriptions are mainly about TPA and TPA-induced TPP. Sequential and simultaneous absorption are two types of TPA. In the former, there is an actual intermediate state between the two photons being absorbed. The absorption of photons at a particular wavelength by a material produces an actual intermediate state, which means it is a surface effect and follows the Beer-Lambert law (Jacobs, 1992). In the simultaneous absorption, on which the TPP is based, a notion of virtual state (not a real intermediate energy state) is often used. It implies that the material is transparent at that wavelength (Farsari et al., 2010). Absorption of the first photon results in a super transient (∼10^−16^ s) virtual state and TPA happens only if another photon arrives within this time (Malinauskas et al., 2013). For this three-order nonlinear optical phenomenon to occur, high light intensities provided by a tightly focused laser are necessary. That is why TPA was predicted by Göppert-Mayer in the 1929 but demonstrated by Kaiser in 1960s benefiting from the invention of the laser. Maybe we can define this high intensity causing TPA as the “threshold of TPA”. As can be seen in Figure 1, an energy gap is closed up by combination of two photons to cause the electron transition and the difference between the two types is whether there is a real intermediate energy state. In addition, TPA can also be divided into degenerate and nondegenerate cases according to whether the energy of the two photons is equal or not (hν1 = hν2 or not). The degree of TPA or the electron transition probability scales as the square of incident light intensity of the laser beam (Rumi and Perry, 2010), which guarantees TPA only occurs in the focus of the beam and the probability rapidly diminishes away from the focal plane. Specifically, by regulating the incident laser power, excitation can be limited to a small region within the focal volume, and the energy outside this region passes through the material without giving rise to any light-material interaction. High light intensities are beneficial to effective excitation of the process, as a consequence, femtosecond lasers with high peak intensities are conventionally used in TPA (Ovsianikov et al., 2012).

TPA is the fundamental process of making TPP. After absorbing energy from the two photons, a PI molecular is excited from the ground state to the excited singlet or triplet state. Some of the excited initiators (Norrish type I) are cleaved from the excited triplet state and produce reactive intermediates to initiate polymerization; some of the excited initiators (Norrish type II) can abstract hydrogen from hydrogen donor and undergo a photo-induced electron transfer and fragmentation process to generate reactive intermediates (Nguyen et al., 2005; Wu et al., 2006). The reactive intermediates include radicals or cations that initiate radical or cationic photopolymerization, respectively. For biomedical scaffolds fabrication, however, due to generating protonic acids that affect cell cultures, cationic initiators are generally avoided (Nguyen and West, 2002), in the following, we only discuss radical-induced polymerization. There is a threshold for any nonlinear process (Jonušauskas et al., 2018), and TPP is no exception. The polymerization by TPA only proceeds when the intensity I is more than the threshold I_th_ of a specific polymerization required by the photopolymerizable materials and other processing steps (e.g. development). By adjusting the light intensity at the focus volume in a manner such that the light-produced radicals initiate polymerization only in a region where I exceed I_th_, the diffraction limit, which has a fixed value for a particular optical system, no longer determines the size of the voxel (Farsari et al., 2010). It should be pointed out here that the threshold of TPA is different from that of the TPP process, which has not been clearly distinguished in most other literature.

**FIGURE 2:**
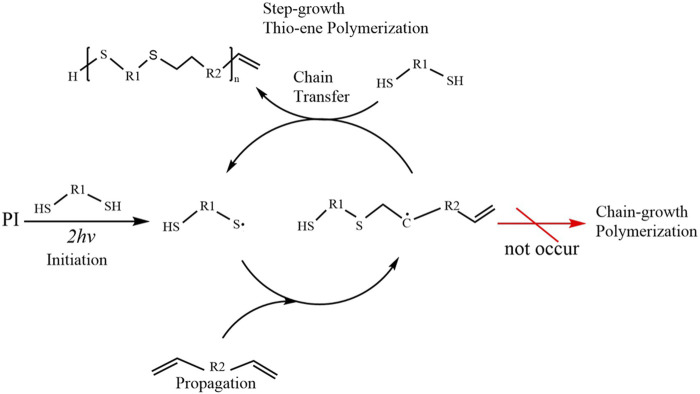
Simplified scheme of two-photon induced polymerization mechanism using thiol-ene photo-click chemistry.

**FIGURE 3:**
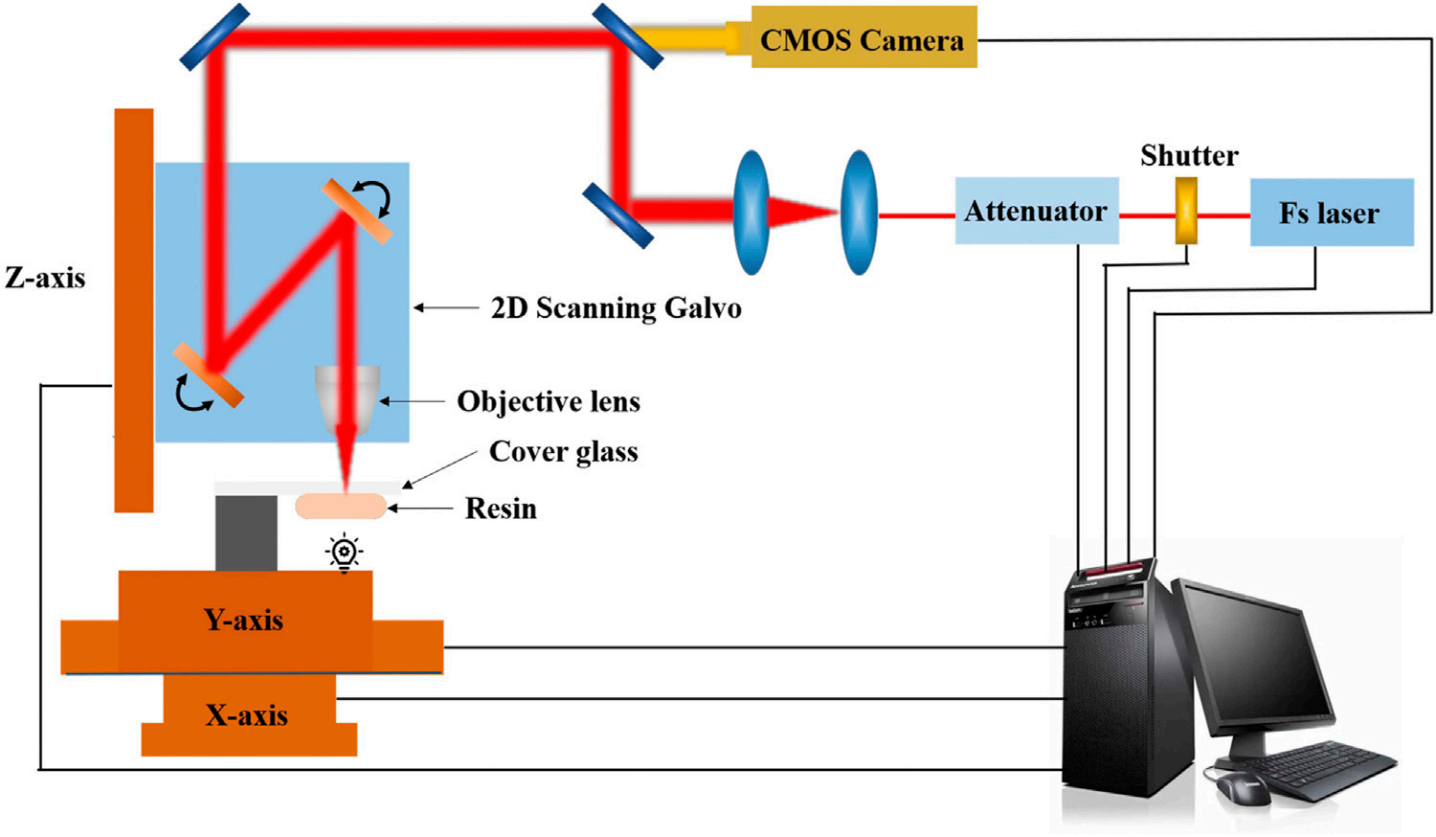
A typical experimental workstation for TPP in our lab.

In addition to the traditional free-radical chain-growth polymerization, the applications of TPP to the step-growth polymerization based on thiol–ene photo-click chemistry (Figure 2) have also been investigated (Qin et al., 2014b). The laser source for TPP is typically from a Ti:Sapphire femtosecond laser with 780–800 nm wavelengths, however, some other femtosecond lasers (e.g., Yb-based femtosecond lasers) and some picosecond lasers are also available for TPP (Malinauskas et al., 2010). The typical experimental workstation for TPP in our lab is given in Figure 3. A mode-locked Ti:sapphire oscillator with a repetition rate of 80MHz, a wavelength of 800nm, and a pulse duration of 100 fs, is used for TPP. The laser beam passing through the attenuator, beam expander, beam splitter and other optical components is tightly focused into the photopolymerizable materials with the oil-immersion objective lens (100×, NA = 1.3) filled with a refractive-index-matching oil (n_oil_ = 1.518). Photopolymerizable materials are scanned by the laser focus in 3D space and polymerization occurs along the trace of the focus. After fabrication of the required structures, the samples must be developed to wash off the unpolymerized materials.

## Materials for two-photon polymerization in biomedical applications

Initially, TPP was mainly used in the field of nanophotonics and achieved a lot of results (Soukoulis and Wegener, 2011; Ovsianikov et al., 2012). However, due to its high-resolution, well-defined geometric structures, and true 3D writing ability, the applications of TPP in the biomedical field have never stopped and the biomaterials for TPP are continuing to extend.

### Photoinitiators

For a photopolymerization process, PI is the prerequisite since it is critical to the processing resolution and efficiency of TPP. Because of the lack of TPP-specific PIs, PIs for single-photon polymerization such as TPO-L, Irgacure 369 were initially used in TPP process (Maruo et al., 1997b; Jhaveri et al., 2009; Ovsianikov et al., 2010). Such initiator molecules usually have a lower TPA cross-section (δ_TPA_<40 GM), which makes them of limited initiation efficiency, therefore, relatively long exposure time and high laser energy were required to achieve polymerization, especially for the typical wavelength of 800 nm.

Although a fully understanding of the relationship between molecular structure and TPA properties poses a challenge to researchers, strategies for the development of TPP-PIs have been proposed. Its main principles are as follows 1) achromophoric groups with large δ_TPA_; 2) a chemical functionality with high initiation efficiency (i.e. high radical yield) and 3) a mechanism of chemical function activation by chromophores excitation (Albota et al., 1998; Kuebler et al., 1999; He et al., 2008). Based on the above strategies, researchers have obtained a large number of highly effective initiator molecules, such as styrene (Kuebler et al., 2001), anthraquinone (Xing et al., 2007; Xing et al., 2012), fluorene (Jia et al., 2019), carbazolyl (Yang et al., 2014) derivatives. However, TPP-PIs with a large δ_TPA_ and high initiation efficiency are not sufficient for the biomedical applications, especially for the field of tissue engineering and hydrogels. The application of PIs in biomedical field calls for good biocompatibility, acceptable water solubility and low cytotoxicity.

Currently, there are not many 2PP-PIs can be used in the biomedical field that meet all the above conditions. In type I PIs, DAS (tetrapotassium4,40-(1,2-ethenediyl) bis(2-(3-sulfo-phenyl)diazenesulfonate)) (Tromayer et al., 2018) is currently the only one that can be applied for cell encapsulation in the process of TPP, although it has a low δ_TPA_ (40 GM at 800 nm), which leads to a low initiation efficiency for TPP (Van Hoorick et al., 2019). Since reactive oxygen species are harmful to cellular function, type I PIs designed to easily cleave are not the most biocompatible class of materials (Nguyen and Narayan, 2017). Thus, more research efforts were geared toward the development of type II PIs for biomedical applications, among which WSPI(1,4-bis [4´-(N,N-bis [6´[bis [trimethylammoniumiodide-6-hexyl]-aminohexyl]amino)styryl]-2,5-dimethoxybenzene)[63], π-Expanded Ketocoumarins (Nazir et al., 2014), P2CK(sodium 3,30-(((1E,1E0)-(2-oxocyclopentane-1,3-diylidene)bis (methanylydiebe))bis (4,1-phenylene))bis (methylazanediyl))dipropanoate) (Li et al., 2013) and T1-T3 (a series of water-soluble benzylidene cyclanone dyes) (Huang et al., 2017) are representative. On account of its facile synthesis (2 steps) and high initiation efficiency (δ_TPA_ = 140 GM at 800 nm), P2CK is a more commonly applied bio-initiator for TPP in recent years. It should be noted that despite P2CK being a biocompatible PI, it is not suitable to be processed together with cells during laser irradiation, since it can penetrate the cell membrane and in the presence of laser irradiation produce singlet oxygen that is toxic to cells (Ovsianikov et al., 2014; Van Hoorick et al., 2019).

### Photopolymers

**FIGURE 4:**
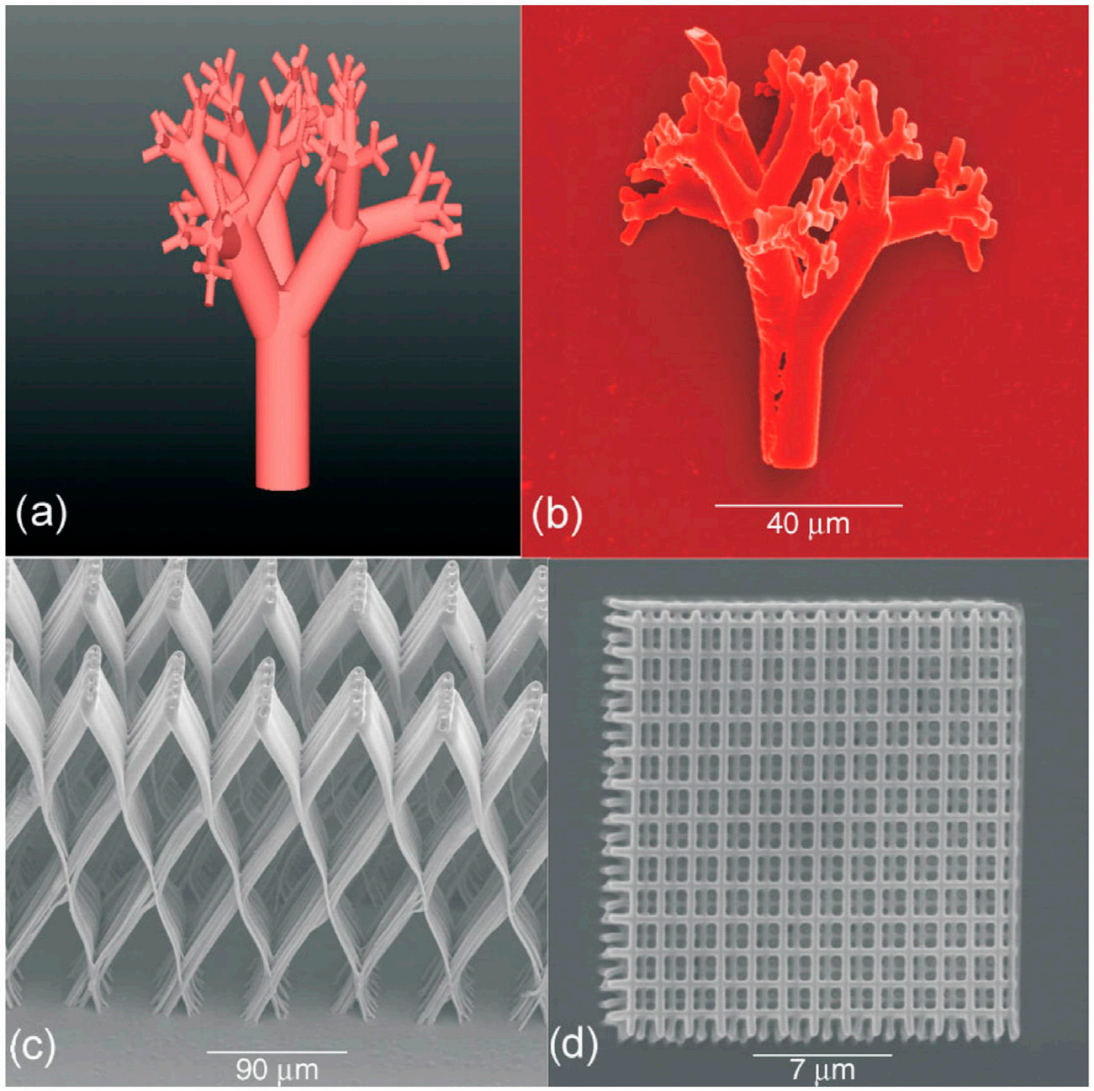
CAD model **(A)** and SEM image **(B)** of a 3D vascular microcapillary structure fabricated by two-photon polymerization technique of SU8. **(C,D)** SEM images of porous structures fabricated from Ormocomp. Reprinted with permission from Reference (Ovsianikov et al., 2007b).

At present, general and commercially available photopolymers mainly include (meth)acrylate-based materials (e.g., PEGDA, ORMOCERs, SZ 2080) and epoxy-based photoresists (e.g., SU8), among which epoxy-based photoresists are cationic polymerization (LaFratta et al., 2007). The first porous 3D microstructures fabricated by TPP were completed using SU8 and ORMOCOMP (a member of the ORMOCERs) in 2007 (Figure 4) and demonstrated the biocompatibility and not cytotoxic of the structures (Ovsianikov et al., 2007b). A few years after that, complex 3D structures fabricated by TPP were mainly made of commercially available (meth)acrylate-based photopolymers such as SR368andSR499 (Tayalia et al., 2008), SI10 (Hsieh et al., 2010), ORMOCERs (Weiß et al., 2009; Klein et al., 2010; Käpylä et al., 2012; Kiyan et al., 2012; Turunen et al., 2017) and PEGDA (Ovsianikov et al., 2007a; Ovsianikov et al., 2011c; Weiß et al., 2011; Nguyen et al., 2013). These studies fully demonstrated that TPP can be used as a potential tool for fabricating complex 3D microstructures that properly mimic natural ECM with the features at micro/nanoscale. Until recent years, commercial photopolymers were still often used to fabricate 3D biomedical scaffolds using TPP, for example, IP-L780 (Nanoscribe GmbH, Germany) which contains more than 95% of pentaerythritol triacrylate and less than 5% of 7-(diethylamino)-3-(2-thienylcarbonyl)-2H-1-benzopyran-2-one is a biocompatible solvent-free photopolymer formulation, developed specifically for TPA, can produce structures with superior spatial resolution, low stress, high mechanical stability and little shrinkage (Paun et al., 2018). The feature sizes of constructs fabricated of IP-L 780 by TPP can be down to 150 nm without any additional procedures (Mihailescu et al., 2016). SZ2080 contains a hybrid organic-inorganic silicon–zirconium composition (Ovsianikov et al., 2008), of which lower shrinkage than ORMOCERs can effectively avoid structural deformation during manufacturing (Ovsianikov et al., 2008; Raimondi et al., 2012). Poly (ethylene glycol) diacrylate (PEGDA), a hydrogel material, is one of the most commonly used photopolymerizable synthetic materials for TPP and is widely used for biomedical applications (Ding et al., 2013; Qin et al., 2014b; Liu et al., 2022).

The above materials all show biocompatibility, however, they are non-degradable materials and are not suitable for the temporary support required for most tissue engineering. Although researchers had developed bioerodible PEG hydrogels using several strategies (Lu and Anseth, 2000; Gobin and West, 2002; Bryant et al., 2004), biodegradable synthetic material first successfully fabricated by TPP for 3D scaffolds was the triblock copolymer poly(ε-caprolactone-co-trimethylenecarbonate)-b-poly(ethylene glycol)-b-poly(ε-caprolactone-co-trimethylenecarbonate) (Claeyssens et al., 2009). The hydroxyl end groups of this triblock copolymer were further modified with methacryloly chloride to produce a photopolymer carrying photopolymerizable methacrylate moieties. Other TPP processable degradable synthetic biopolymers such as poly (lactic acid)-, urethane-,oligolactones-, and poly(trimethylenecarbonate)-based photopolymers have been demonstrated (Melissinaki et al., 2011; Weiß et al., 2011; Timashev et al., 2016; Weisgrab et al., 2020). It should be pointed out here, using methacrylated groups as photopolymerizable moieties to modify the materials to make them photopolymerizable is also the formation strategy of many photopolymers, including naturally-derived biomaterials. The photoreactivity of acrylates is superior to methacrylates in TPP applications, however, irritation and toxic effects of residual acrylate groups pose a risk for long-term clinical safety (Qin et al., 2014b). In contrast, methacrylates widely used as dental filling materials possess much less cytotoxic (Moszner and Hirt, 2012). Although there is no scaffold fabrication, poly (ethylene glycol) dimethacrylate (PEGDMA), a TPP processable material, instead of PEGDA, has been used to fabricate 3D controlled drug delivery devices (Do et al., 2018). It is noteworthy to mention that vinyl ester- and vinyl carbonate-based photopolymers with higher biocompatibility and without cytotoxic degradation products have been reported (Heller et al., 2009; Mautner et al., 2013; Qin et al., 2014a; Mautner et al., 2016). Furthermore, the medium photoreactivity of vinyl esters has been extremely ameliorated by the utilization of thiol-ene chemistry (Mautner et al., 2013; Qin et al., 2014a).

Given that natural materials may have a natural affinity for surrounding cells, it makes sense that naturally-derived biomaterials are often thought to have an advantage over synthetic ones (Qin et al., 2014b). Most naturally-derived biomaterials either originated from natural ECM or possess properties similar to those of the natural cellular environment (Qin et al., 2014b). A variety of naturally-derived materials have been studied for TPP, including gelatin, chitosan (CH), hyaluronic acid (HA), alginate, and so forth. As early as 2009, CH has been combined with triacrylate monomers as a natural material to fabricate 3D scaffolds using TPP, simply retaining CH as a dopant within the structure without forming any kind of crosslinking (Correa et al., 2009). Gelatin derived from collagen Type I is the main component of the natural ECM of mammals (Van Hoorick et al., 2019), containing tripeptide arginine-glycine-aspartic acid (RGD) and matrix metalloproteinase (MMP) sequences in the protein backbone conduce to cell-interactive and enzymatically degradable properties (Bello et al., 2020). Gelatin-methacryloyl (Gel-MA) or methacrylamide-modified gelatin (Gel-MOD) which is formed by reacting the primary amines of hydroxylysine, lysine and ornithine with methacrylic anhydride inherits the bioactivity and biodegradability of gelatin (Van Hoorick et al., 2019; Piao et al., 2021), as a consequence, Gel-MA has become one of the gold standards in the biomaterial realm (Van Hoorick et al., 2019). With the utilization of GEL-MA, TPP made important progress in processing naturally-derived photopolymers (Ovsianikov et al., 2011a). HA, CH and alginate are all linear, hydrophilic polysaccharides with outstanding biocompatibility, among which HA is an important component of natural ECM (Qin et al., 2014b). Photopolymerizable hyaluronic acid can be generated using glycidyl methacrylate-based modification to obtain HA-glycidyl methacrylate conjugates, which have been demonstrated to be suitable for the fabrication of 3D porous scaffolds via TPP (Kufelt et al., 2014). Photopolymerizable CH and alginate generated using a similar strategy have also been demonstrated (Kufelt et al., 2015; Garcia-Lizarribar et al., 2018). These naturally-derived biomaterials are all hydrophilic monomers/macromers, which can form hydrogels through cross-linking networks. Through the precise design of its physical and chemical properties and biological characteristics, hydrogels can highly simulate the natural ECM environment *in vitro* and reasonably regulate the life activities of cells and the process of tissue regeneration (Qin et al., 2014b). In addition, due to the minimal damage to cells when used for cell culture (Cushing and Anseth, 2007), they are ideally suited for the fabrication of 3D biomedical scaffolds. The biodegradability issue of naturally-derived photopolymers will be discussed separately in the following section.

**FIGURE 5:**
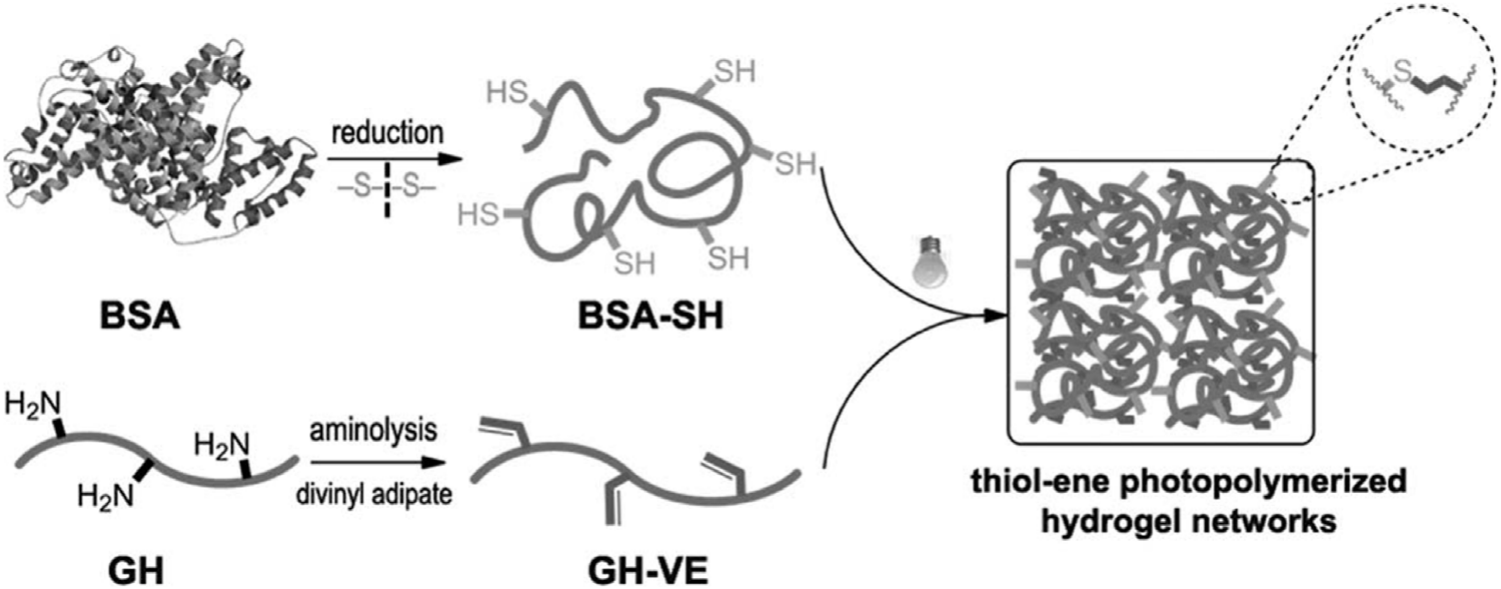
Hydrogel formation by thiol-ene photopolymerization using GH-VE and BSA-SH. Reprinted with permission from Reference (Qin et al., 2013).

All of photopolymers for TPP mentioned thus far have taken advantage of a chain-growth polymerization approach. Free-radical chain polymerization is a facile mechanism because of straightforward material handling. Furthermore, the introduction of methacrylate groups to the target materials involves single or several relatively simple steps reactions resulting in substantial applications as mentioned above. However, chain growth has its inherent disadvantages, including 1) the formation of a more heterogeneous network due to the presence of a multitude of kinetic chains; 2) a diminished control over the number of reacted functionalities due to the more complicated kinetic profile of free radical chain-growth polymerizations; 3) the demand for higher PI concentrations in combination with higher spatiotemporal energy due to oxygen inhibition during polymerization (this results in longer processing times) (Hoyle and Bowman, 2010; Pereira and Bártolo, 2015; Van Hoorick et al., 2020). Accordingly, radical-mediated thiol–ene photo-click chemistry (Figure 2) is based on the exceptionally efficient reaction of thiols with non-homopolymerizable C-C double bonds, leading to a step-growth polymerization for a network formation, has become another important research direction (Lowe et al., 2010). Chemical reactions with thiol–ene click chemistry which occurs very fast under relatively mild conditions, are particularly well-suited for polymerization reactions and require minimal radical initiating species, with the absence of any side products and highly controlled high yield, forming crosslinked polymer networks with a high degree of homogeneous structures, are extremely suitable for extensive precursors containing numerous ene or thiol groups and ideal to biomedical applications (Lowe et al., 2010; Van Hoorick et al., 2019). In summary, step-growth polymerization based on photo-click chemistry can overcome the disadvantages of chain-growth polymerization. In 2013, TPP experiments for 3D biomedical scaffolds were explored for the first time using thiol-ene photo-click chemistry (Qin et al., 2013). Although methacrylate-based monomers are generally used in TPP processing, it is believed that the reactivity of methacrylates cannot be improved by using thiol-ene strategy. As a consequence, using vinyl ester derivative of gelatin hydrolysate (GH-VE) and reduced bovine serum albumin (BSA-SH) (as thiols crosslinker) (Figure 5), the experiments proved that TPP can fabricate hydrogel microstructures with superior definition and stability at fairly high throughput (50 mms-1 scanning speed) *via* photo-click chemistry approach (Qin et al., 2013). Quite a number of different thiol/ene modified materials have been successfully employed with TPP for biomedical 3D structures, including thiol-ene modified poly (vinyl alcohol), ene-functionalized gelatin, thiol-ene modified recombinant protein, and thiolated gelatin (Baudis et al., 2016; Van Hoorick et al., 2018; Tytgat et al., 2020; Van Hoorick et al., 2020). It is important to point out that naturally-derived materials have problems with product variability as well as the risk, albeit small, of the immune response. To address the above concerns, an interesting photopolymer, methacrylamide-modified recombinant peptide, has been developed for TPP to fabricate tissue engineering scaffolds (Tytgat et al., 2020). A recombinant peptide (RCPhC1) which is based on human collagen type I and enriched with RGD tripeptide sequences is highly reproducible and contains no animal-derived components (Tytgat et al., 2019). Methacrylamide-modified RCPhC1 (RCPhC1-MA) was first developed according to the protocol for Gel-MA in 2019 as photopolymerizable collagen mimics but no TPP experiment was conducted on it at that time (Tytgat et al., 2019). A year later, the same research group went on to synthesize RCPC1-MA, norbornene-modified RCPhC1 (RCPhC1-NB) and thiolated RCPhC1 (RCPhC1-SH) (Tytgat et al., 2020). TPP processing and cell encapsulation assays showed that RCPhC1-NB/SH hydrogels have excellent biocompatibility and processability for TPP.

Although the above discussion of photopolymers follows the sequence of commercially available, synthetic, and naturally-derived materials, it is not a classification of materials science. For example, PEGDA is a synthetic material while SZ2080 belongs to hybrid organic-inorganic materials. In practice, various materials are frequently combined to fabricate 3D structures *via* TPP to tune the relevant mechanical and biochemical properties. As mentioned above, CH was doped with triacrylate monomers to fabricate 3D scaffolds (Correa et al., 2009). Scaffolds fabricated of blended materials by TPP, such as a mixture of pentaerythritol triacrylate (PETA) and bisphenol A glycidyl methacrylate (BisGMA) have been also reported (Heitz et al., 2017). Here is necessary to discuss hybrid organic-inorganic photosensitive materials as it is a widely investigated class of materials for TPP (Farsari et al., 2010). As we all known, TPP initially was mainly used in the field of photonics while ORMOCERs which is a silicate-based organic-inorganic hybrid material were once the most widely used in TPP, however, its shrinkage during the structuring and development procedures led to the aberration of the crystals and the disappearance of the photonic band gap poses a challenge to TPP processing (Ovsianikov et al., 2008; Farsari et al., 2010). As a consequence, a research group of Ovsianikov investigated the material combination of silicon and zirconium alkoxides and used zirconium propoxide doped in silicon alkoxide to develop a non-shrinking Zr-based hybrid organic-inorganic photopolymer through sol-gel organic-inorganic hybrid technology, which is also the material combination of commercially available SZ 2080 (Ovsianikov et al., 2008; Farsari et al., 2010). To date, using the same technology and similar strategies, organic-inorganic photopolymer incorporating Ge, Ti, V, and Al have been produced for TPP (Sakellari et al., 2010; Malinauskas et al., 2011; Kabouraki et al., 2013; Balčiūnas et al., 2019), among which Ti-, V-and Al-based hybrid materials have been used for the fabrication of tissue engineering scaffolds using TPP have been demonstrated to be biocompatible (Psycharakis et al., 2011; Balčiūnas et al., 2019).

## 3D Scaffolds fabricated *via* two-photon polymerization

Investigating cytobiology and tissue physiology and pathophysiology outside of the organism needs an *ex vivo* defined cell culture platform. Cell functionality, such as proliferation, differentiation, migration, and adhesion, responding to the physicochemical and biological characteristics of the surrounding environment differ considerably in physiological 3D environments from those in 2D tissue culture plastics (TCP) (Tibbitt and Anseth, 2009; Tibbitt and Anseth, 2012; Vu et al., 2015). Or we could argue that traditional cell culture systems developed for cytobiology cannot felicitously replicate the function and structure of real biological tissue. For example, a pioneering work demonstrated that cultured on a 2D substrate mammary epithelial cells display cancerous phenotypes while in a 3D environment they develop into a normal acinus structure (Petersen et al., 1992). An *in vitro* culture system is critical to the understanding of the liver disease, including the progression and repair mechanisms. When liver cells are cultured on 2D TCP, abnormal proliferation and loss of hepatic function will occur. As a consequence, cell culture has shifted from 2D platforms to 3D microstructures (Dutta and Dutta, 2009; Lau et al., 2012; Lin et al., 2014). It is worth mentioning here that stem cells are a special kind of cell, that promise to provide unlimited amounts of cells for transplantation, and has been the focus of regenerative medicine in recent years (Akhmanova et al., 2015). Stem cells reside in a stem cell niche that is a specialized microstructure to control stem cell growth and differentiation by imparting biochemical and biophysical cues (Tibbitt and Anseth, 2012; Akhmanova et al., 2015).

Human tissues consist of a complex organization of cells, ECM, and signaling molecules. *In vivo*, they are composed of an arrangement of iterative basic units in the size of 100–1000 μm (Mikos et al., 2006; Atala et al., 2012). Tissue engineering scaffold, acting as an ECM, interacting with cells prior to forming new tissues (Li et al., 2016; Jafari et al., 2017; Zhang Q et al., 2019; Xue et al., 2020), is the crus of a classic tissue engineering approach (Langer and Vacanti, 1993). It has been identified as potential element that forms the basic concepts of regenerative medicine and is considered to be central to the development of regenerative therapy (Ratheesh et al., 2017; Wu et al., 2020). In order to provide an engineering ECM for cells to grow, proliferate, and differentiate to form new tissues, scaffolds have to possess suitable properties, including biocompatibility, degradability (for most of the tissue engineering) at a suitable rate, adequate mechanical properties and high porosity and pore interconnectivity (Zhang S et al., 2019). In addition to the above basic requirements, integrating different properties and cues such as hydrophilicity, biological and physical cues (e.g., various functional groups, structure and surface morphology and so forth) could facilitate the formation of new tissues (Atala et al., 2012; Higuchi et al., 2013; Jafari et al., 2017).

TPP is a distinctive and powerful approach to implement additive manufacturing for the realization of 3D biomedical scaffolds due to its ability to fabricate artificially designed constructs with arbitrary geometry on the cell or sub-cellular size scale comparable to that found in many human tissues (Nguyen and Narayan, 2017; Baldacchini et al., 2021). Next, we will discuss the typical 3D architectures produced by TPP for applications in cell biology, tissue engineering and regenerative medicine from the perspective of engineering according to according to different material categories.

### Scaffolds fabricated of commercially available photoresist

The earliest material used to manufacture tissue engineering scaffold using TPP is a commercial photopolymer ORMOCOMP, which is a member of the ORMOCERs (Ovsianikov et al., 2007b; Schlie et al., 2007). In the experiments, researchers demonstrated for the first time the huge potential of TPP for fabrication of 3D biomedical scaffolds with rationally designed topology, however, the resolution of the structures was not characterized in details. Soon after, researchers used viscous triacrylate two-monomer composition (SR368andSR499) by TPP to fabricate the first cell culture scaffold with different lateral pore sizes (12–110 µm) to studied cell migration in the scaffolds using human fibrosarcoma cell line (Tayalia et al., 2008). This experiment demonstrated the ability of TPP to precisely control the pore size of 3D matrix. In 2009, Weiß et al. utilized ORMOCER to manufacture 3D biological scaffolds using 2PP and characterized the resolution of the structures (Weiß et al., 2009). Using a 40×objective lens with lower numerical aperture (NA 0.6) at wavelength of 800 nm for fast fabrication of larger scaffold structures, they obtained woodpile structures with lateral and axial feature sizes of about 5 and 20 µm. Meanwhile they demonstrated a miniaturized copy fabricated using a 100×objective lens (1.4 NA) with a spatial resolution of about 500 nm.

**FIGURE 6:**
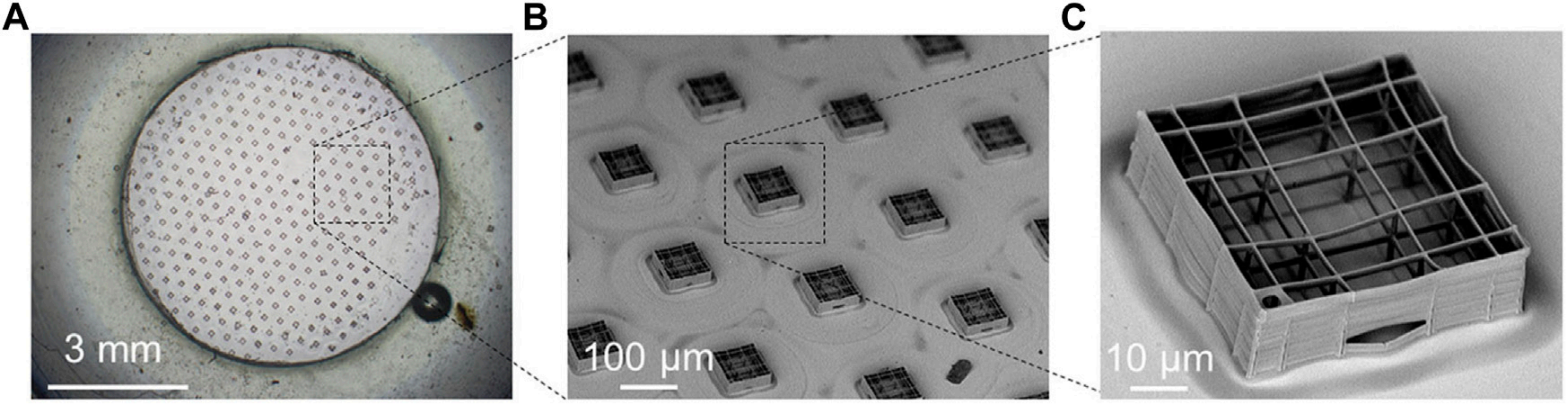
The improved synthetic niche culture system fabricated by two-photon laser polymerization (TPP). **(A)** Optical microscope image of the TPP substrate. The number of engineered niches was increased from 7 to around 400 niches per sample, covering 10% of the available culture surface to obtain larger niche-cultured cell numbers to perform quantitative analysis and functional assays. The relative distance between niches has been set at 300μm. **(B)** Scanning electron microscopy (SEM) image of the synthetic niches forming 2PP substrates. **(C)** SEM image of a single synthetic niche. Reprinted with permission from Reference (Nava et al., 2017).

**FIGURE 7:**
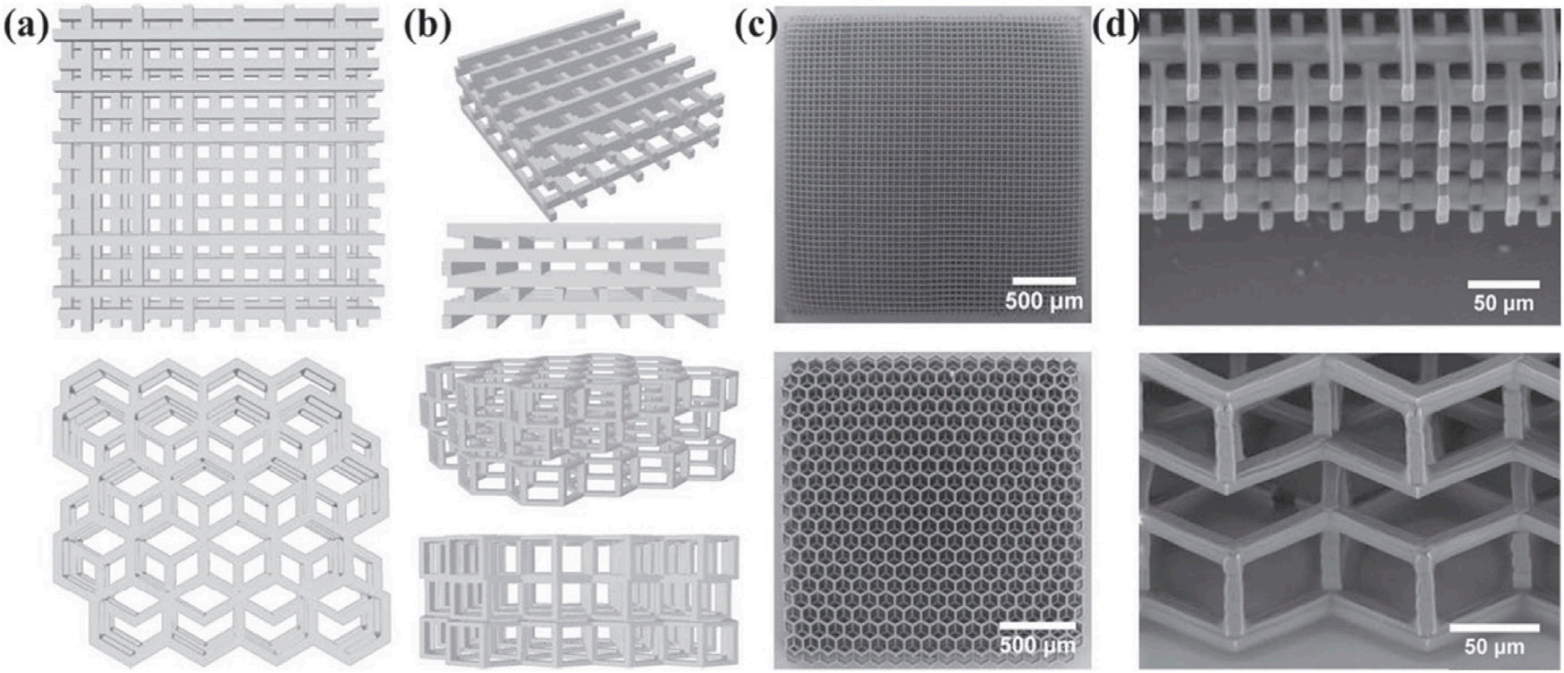
Scaffolds fabricated for cartilage tissue engineering *via* TPP. Schematics of the fabricated 3D SZ2080 scaffolds: the top line corresponds for the woodpile scaffold, and the bottom line corresponds for hexagon geometries, respectively. **(A)**,**(B)** CAD models (top and oblique), **(C)**,**(D)** SEM micrographs (top and oblique). Reprinted with permission from Reference (Maciulaitis et al., 2015).

Another market available biocompatible photopolymer commonly used by TPP is SZ 2080. Employing SZ 2080, Raimondi et al. used 100×objective lens with 1.4 NA at wavelength of 800 nm to fabricate artificial stem cell niches with two heights (20 and 80–100 µm) and four lattice pore dimensions (10, 20, 30 μm and graded) to investigate the effects of purely structural cues on stem cell behaviors (Raimondi et al., 2013). In fabricating the niches, scanning speeds 60, 10, 2 and 2 μm s ^−1^ corresponded to 10 μm, the graded, 20 and 30 µm pore sizes,respectively. After determining the optimum geometry for mesenchymal stem cells (MSCs) homing and proliferation, this group fabricated a new substrate with approximately 400 TPP niches on it to investigate the influence of the substrate on MSC proliferation and differentiation (Nava et al., 2017) (Figure 6). SZ2080 stem cell niches coated with thin layers of HA-based and gelatin-based hydrogels also were made using TPP to investigate the interactions between structural and chemical biomimetism on the response of stem cells (Nava et al., 2015). In addition, 3D cartilage tissue scaffolds made of SZ2080 by TPP (Figure 7) have been in preclinical study (Maciulaitis et al., 2015). Gaining popularity in recent years, IP-L 780 was used to fabricate porous 3D cell-seeding constructs for bone tissue engineering *via* TPP (Mihailescu et al., 2016). To search for the optimum laser parameters to obtain the ideal structures, various combinations of the laser power varied from 20 to 44 mW and the scanning speed between 50 and 100 μm s^−1^ were tested in the experiment. In another investigation of complex stackable scaffolds by TPP for spatial organization of living cells (Larramendy et al., 2019), Larramendy et al. confirmed that using a 780 nm wavelength laser of 11 mW by means of a 100×objective lens (1.4 NA) at the scanning speed of 30 μm s^−1^ results in a lateral width of polymerized line of about 0.3 μm and an axial thickness of about 1μm.Recently, to investigate the response of macrophages,Nouri-Goushki et al. used Photonic Professional GT machine (Nanoscribe, Germany) to print six different patterns of micropillars made of IP-L 780 (height = 250, 500, 1,000 nm, diameter = 250 nm, and interspacing = 700, 1,000 nm)) *via* TPP at a relatively high scanning speed of 1,200 μm s^−1^ ( Nouri-Goushki et al., 2021). This reflected IP-L 780 optimized sensitivity for fast 3D structuring. As can be seen from the above examples, the relevantly geometric or mechanical properties of the scaffolds can be achieved by adjusting the laser parameters and scanning speed, which obviously belongs to the manufacturing strategy of mechanical engineering.

### Scaffolds fabricated of synthetic materials

As the most broadly used synthetic hydrogel material in the biomedical field, PEGDA is frequently applied in TPP 3D scaffolds processing (Ovsianikov et al., 2007a; Ovsianikov et al., 2011c; Klein et al., 2011; Weiß et al., 2011; Nguyen et al., 2013; Accardo et al., 2018a; Yu et al., 2019; Song et al., 2021). In view of the bioinertia and non-degradability of PEGDA, by incorporating proteolytically degradable peptide sequences into the backbone and further modifying the hydrogels with cell adhesive ligands, hydrogels consist of PEGDA can be rendered bioactivity and biodegradability (Gobin and West, 2002; Lee S. H. et al., 2008). Using pentaerythritol tetraacrylate (PETTA) or PETA as crosslinker, the disadvantage of its water binding properties causing distortion and partial loss of geometry control can be overcome to improve the stability of 3D scaffolds (Klein et al., 2011; Yu et al., 2019). Accardo et al. utilized TPP to fabricated 3D hydrogel scaffolds that has the true free-standing nature to allow an efficient colonization of neuronal cell line neuro2A (Accardo et al., 2018b). Further, low fluorescent emission feature of PEGDA ensures an immunofluorescence 3D characterization by two-photon confocal imaging for the microexamination of neuronal cell growth (Accardo et al., 2018b). Recently, Song et al. selected PEGDA as cell-repellent photoresist in conjunction with a methacrylated recombinant peptide (RCP)-based photoresist as the cell-adhesive photoresist to control cell alignment through 2D and 3D structures with alternating stiffness that was achieved by using alternating TPP laser power (Song et al., 2021). It is should be noted here, the Young’s modulus is often referred to in a biological context simply as stiffness or elasticity (Tse and Engler, 2011), which is different from that in mechanical engineering or materials science.

In addition, PETA can be bio-compatibly used as the main prepolymer combined with other photopolymers to fabricate TPP cells scaffolders (Heitz et al., 2017), while PEGDA can also be used as a crosslinker (Brigo et al., 2017) or one of the components of blend photopolymers to manufacture biomedical scaffolders using TPP (Kufelt et al., 2014). In (Kufelt et al., 2014), the researchers conducted TPP processing experiment on 10 wt% pure hyaluronic acid-glycidyl methacrylate (HAGM) and9:1 wt% HAGM/PEGDA, which showed that the addition of PEGDA increased the TPP scanning speed for 3D hydrogel fabrication from 300 µm s to 1 up to 1,000 μm s^−1^ under the same other conditions. Moreover, the combination of HAGM with different ratios of PEGDA can influent the mechanical properties of the generated gels, materials with a high PEG content indicated high stiffness without affecting the biocompatibility. Combining different materials and adjusting the proportions of components to regulate mechanical properties and processability of product is another manufacturing strategy of mechanical engineering.

Another interesting synthetic photopolymer is star-shaped methacrylate-functionalized poly(D, L-lactide) with four arms. Kuznetsova et al. first used this material to make bone regeneration scaffolds using TPP and confirmed the biocompatibility and biodegradability of the structures (Timashev et al., 2016). Later, they demonstrated that the surface roughness of the scaffolds increased significantly with the extension of lactide arms (Kuznetsova et al., 2017). The scaffolds made of poly(D, L-lactide) with longer arms improved the *in vitro* differentiation of osteogenic MSCs and led to the *in vivo* deposition of calcium phosphate particles.

### Scaffolds fabricated of naturally-derived materials

Gel-MA has been explored to fabricated tissue engineering scaffolds using TPP with and without cells and the structures showed superior cell-interactivity and enzymatic degradability (Ovsianikov et al., 2011a; Ovsianikov et al., 2011b; Ovsianikov et al., 2014), In particular, Prina et al. recently used it to fabricate the precise geometry of the limbal epithelial crypt structures (stem cell “microniches”) seeded with human limbal epithelial stem cells (hLESCs)to study the proliferation and the various differentiation expression of the stem cells, which again demonstrated the outstanding processability of Gel-MA for TPP (Prina et al., 2020). However, post-processing aberrations because of inferior mechanical properties and swelling make the scaffolds very difficult to fully match the CAD model even at relatively high concentrations of Gel-MA (20 wt%), which is a challenge for manufacturing engineering. Some strategies were used to improve mechanical properties. For example PEGDA was co-crosslinked with Gel-MA to form a co-network (Brigo et al., 2017), or the structures were fabricated on a support made of a stronger material (Engelhardt et al., 2011). Particularly, by means of further methacrylation of the carboxylic acids present in Gel-MA, the group of Van Hoorick developed a novel gelatin derivative (GEL-MOD-AEMA) for TPP, which has three times as many photopolymerizable functionalities as Gel-MA and maintains good biocompatibility and biodegradability (Van Hoorick et al., 2017). TPP processing indicated GEL-MOD-AEMA is superior in the aspect of applied laser power (≥ 40 mW (GEL-MOD-AEMA) vs ≥ 60 mW ( GELMA))at 100 mms^−1^) as well as post construct swelling (0–20 % (GEL-MOD-AEMA) vs 75–100% (Gel-MA)). In another study, Mandt et al. used GEL-MOD-AEMA to fabricate biomimetic placental barrier structures via TPP (Mandt et al., 2018). During the structuring process, they adopted a manufacturing strategy to achieve an optimal balance between structure stability and processing time by changing layer distance (dz) and line distance (hatch). Water-insoluble chitosan has also been developed into water-soluble photopolymerizable chitosan hydrogels and used for processing 3D scaffolds by TPP, such as CH-glycidyl methacrylate (CHGM)) (Kufelt et al., 2015) and N-maleyl chitosan methacrylate (MA-CS-GMA) (Parkatzidis et al., 2019). Moreover, Like HAGM mentioned in the previous section, CHGM was combined with PEGDA and MA-CS-GMA with Gel-MA to fabricate TPP 3D scaffolds that showed controllable biological and mechanical properties as CH/PEG and MA-CS-GMA/GELMA composites.

All the TPP scaffolds discussed above were by means of chain-growth polymerization, which remains subject to the disadvantages as discussed earlier. Several years after the first TPP experiment using thiol-ene photo-click chemistry mentioned in 3.1, Van Hoorick et al. used norbornene functionalities to modify gelatin to yield Gel-NB (Van Hoorick et al., 2018). Compared with Gel-MA and Gel-MOD-AEMA, Gel-NB exhibited significantly improved processability in the processing of TPP with dithiothreitol (DTT) as thiolated crosslinker, which was specifically reflected in the minimum laser power at 100 mm s^−1^ scanning speed (20 mW (Gel-NB)vs≥ 60 mW (Gel-MA) vs≥ 40 mW (Gel-MOD-AEMA))and processable concentration range ((≥5 w/v% (Gel-NB)vs ≥ 10 w/v% (Gel-MA and Gel-MOD-AEMA)). A TPP micro-scaffolds for cell culture were fully colonized by fibroblasts after 1 week, demonstrating biocompatibility and potential of 3D processing of the material. Furthermore, a superior CAD mimicry was observed by comparison with GEL-MA due to the significantly lower swelling ratios of Gel-NB structures (Van Hoorick et al., 2018). The following year, the same research group further characterized TTP processing of Gel-NB + DDT system in great detail, including the TPP thresholds corresponding to scanning speed varying between 100 and 1,000 mm s^−1^, swelling ratios of the structures as a function of writing speed and structuring power, relationship between indentation modulus of the samples and TPP processing parameters, different enzymatic degradation rates resulted of different laser powers (at 1,000 mm s^−1^ scanning speed) (Dobos et al., 2020). After that, Gel–NB hydrogel constructs including direct encapsulation, cell-seeded and stiffness gradient scaffolds were fabricated by TPP at an exceptionally high scanning speed (1,000 mm s^−1^) and showed superior biocompatibility, supported cell adhesion and migration (Dobos et al., 2020). In a more thorough research by Van Hoorick group, Gel-NB with a high degree of substitution (DS) (i.e. 90%) benchmarked against Gel-MA with a comparable DS (i.e. 95%) was developed to study the effects of different thiolated crosslinkers on the properties of polymer structures (Van Hoorick et al., 2020). Six crosslinkers applied in the performed assays were DDT, tetraethylene glycol dithiol (TEG2SH), PEG dithiol with a molar mass of 3400 (PEG2SH 3400), PEG tetrathiol with a molar mass of 10,000 or 20,000 (PEG4SH 10000 orPEG4SH 20000) and thiolated gelatin (Gel-SH). TPP processing assay in the study indicated that substantially (i.e. 20-fold decrease) lower polymerization threshold in comparison to the conventional Gel-MA hydrogels could be used to crosslink the thiol-ene systems, taking no notice of the six different crosslinkers. The Gel-NB + Gel-SH combination showed the best mimic for Gel-MA in the aspect of mechanical properties with comparable cytotoxicity and optimal shape fidelity. Even better, the crosslinking network of Gel-NB + Gel-SH possesses the added benefit as it contains only fully biodegradable and bio-interactive components without any residual non-degradable polymer chains.

Building on previous works (Heller et al., 2009; Mautner et al., 2013), Qin et al. explored a series of HA vinyl esters (HA-VE) macromers with tunable DS by lipase-catalyzed transesterification for the fields of biomaterials and tissue engineering (Qin et al., 2014a). HA-VE were proved to be low cytotoxic, fully enzymatically degradable with non-toxic degradation products and suitable for fast TPP processing with µm-scale accuracy via thiol-ene based polymerization. In another further more exploration of HA-VE, different macromer sizes and degree of substitution of HA-VEs were developed in order to study the impact of constitutional parameters of thiol-ene photocrosslinkable hydrogels on material properties. Finally, the formulation 5wt% HA22VE95 (HA with a m. w. of 22 kDa having a DS of 95%)/80 mol% DTT which ensures the high crosslink efficiencies during the fast (100 mm s^−1^ scan speed) 2PP structuring process were used to encapsulate immortalized human adipose-derived mesenchymal stem cells (ASC) in 3D via TPP and their survival monitored for up to 7 days (Zerobin et al., 2020).

As can be seen from the above examples, one of the main bottlenecks of mechanical engineering to improve throughput of TPP is biocompatibility and highly reactive material. Due to hardware limitations, it was usually anticipated to that TPP has a limited throughput resulting from the low scanning speed in the order of less than 1 mm per second in the past many years.

In addition to the materials available for TPP discussed above, non-commercially available hybrid organic-inorganic photopolymer materials are also widely used in biomedical TPP scaffold fabrication (Terzaki et al., 2013; Chatzinikolaidou et al., 2015; Koroleva et al., 2015; Chatzinikolaidou et al., 2017). Koroleva et al. synthesized hybrid organic–inorganic silicon-zirconium photosensitive material using the protocol mentioned earlier (Ovsianikov et al., 2008) and applied it to fabricate 3D porous scaffolds with various pore sizes that were seeded with human bone marrow stem cells (hBMSCs) and human adipose tissue derived stem cells (hASCs) to study the effect of the osteogenic medium and pore size, stiffness, hardness of scaffolds on osteogenic differentiation of stem cells and formation of bone matrix (Koroleva et al., 2015). Terzaki et al. demonstrated a hybrid organic-inorganic material (a blend of hydrolyzed methacryloxypropyl trimethoxysilane (MAPTMS), zirconium propoxide (ZPO), and 2 (dimethylamino)ethyl methacrylate (DMAEMA)) has the excellent biocompatibility, mechanical properties and processability for fabrication of 3D bone tissue engineering scaffolds by TPP (Terzaki et al., 2013). After that, Chatzinikolaidou et al. used this hybrid material to fabricate high-precision TPP 3D scaffolds that were functionalized or disabled by the osteoinductive protein rhBMP-2 and cultured bone marrow derived mesenchymal stem cells (BM-MSCs) on them, the investigations laid the foundation for the potential applications of the cell–material combinations in bone tissue regeneration (Chatzinikolaidou et al., 2015; Chatzinikolaidou et al., 2017).

**FIGURE 8:**
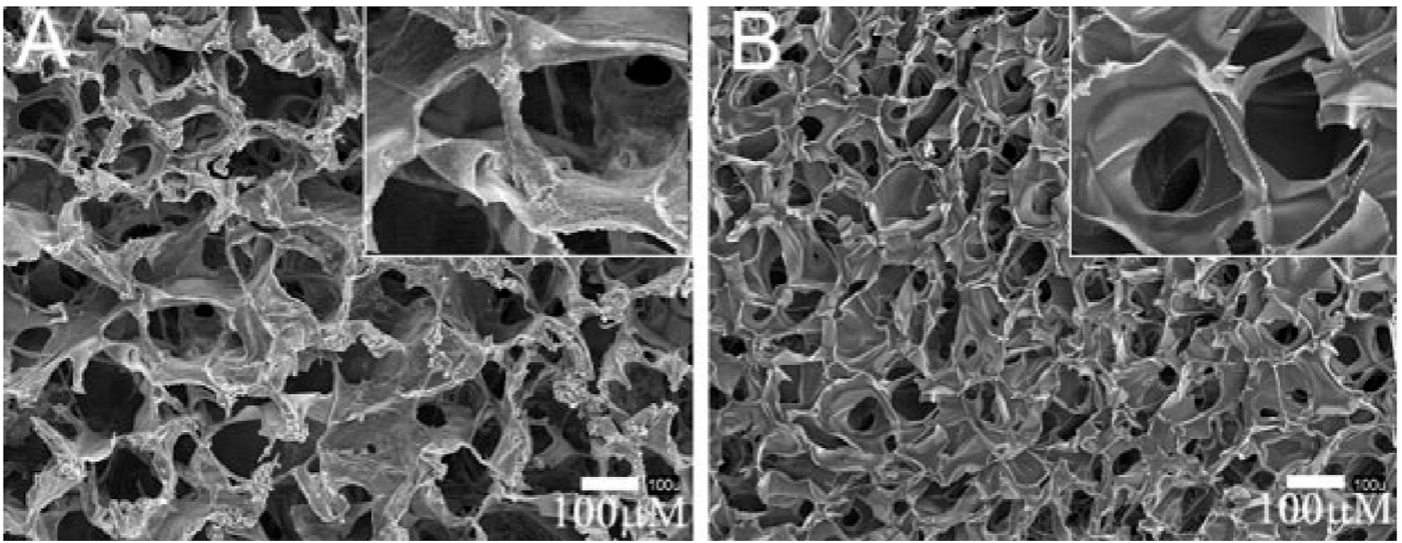
Scaffolds fabricated for cartilage tissue engineering via phase separation followed by freeze drying. SEM images of **(A)** chitosan–alginate and **(B)** pure chitosan scaffolds. The insets detail the pore interconnectivity of the microstructures. Reprinted with permission from Reference (Li and Zhang, 2005).

**FIGURE 9:**
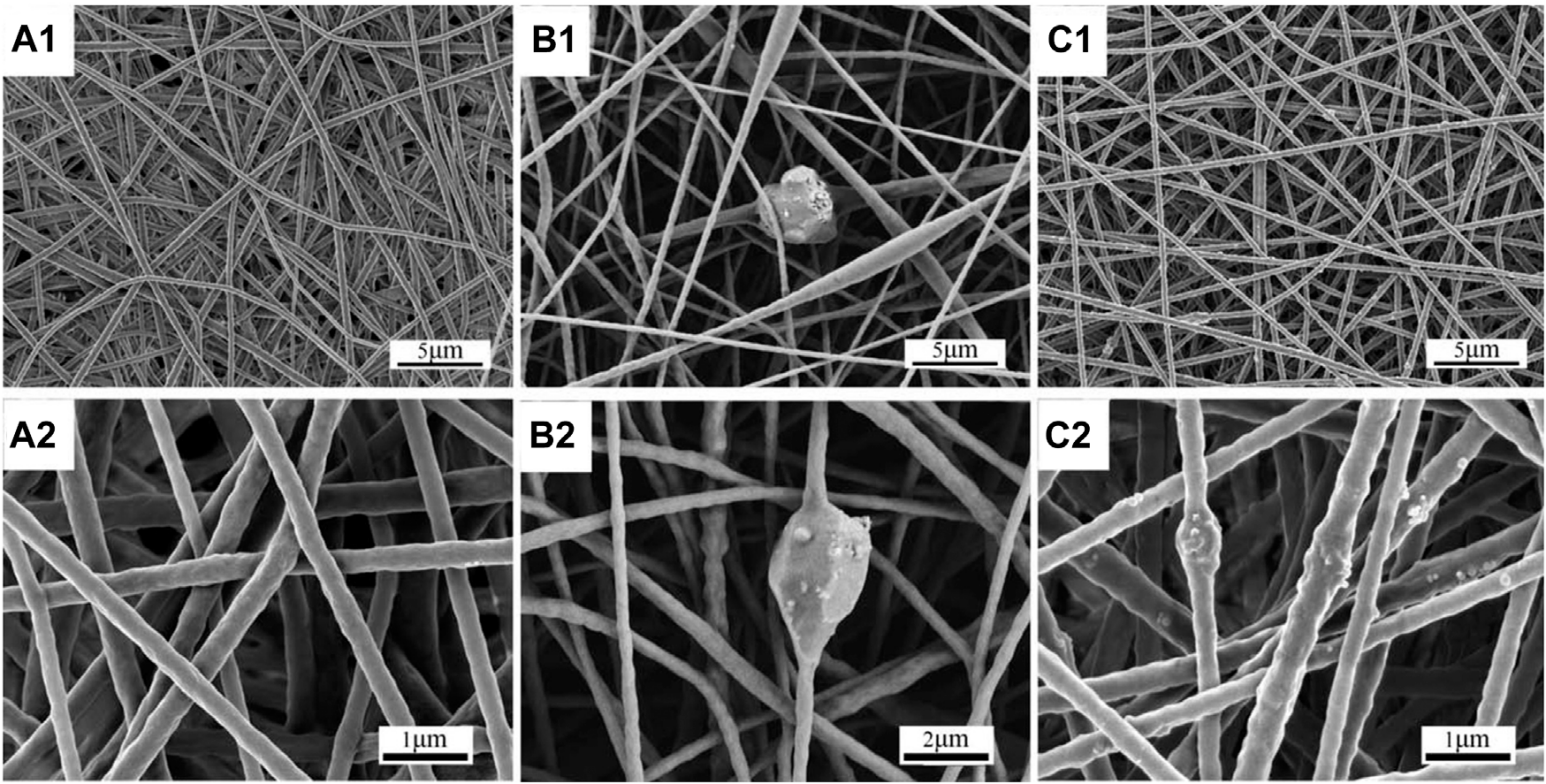
Scaffolds fabricated for bone tissue engineering by electrospinning. Morphology of electrospun hard TPU scaffolds: **(A)** H, **(B)** mH, and **(C)** nH. Images with subscript 1 are at low magnification and images with subscript 2 are at high magnification. Reprinted with permission from Reference (Mi et al., 2014).

At the end of this section, it is necessary to point out that again, as mentioned in the introduction, TPP has unique application advantages over traditional biomedical scaffold manufacturing techniques, which are mainly reflected in the spatial resolution of the fabricated structures and the accurate CAD model replication. Figures 8, 9 show bone and cartilage tissue scaffolds fabricated using phase separation followed by freeze drying and electrospinning techniques (Li and Zhang, 2005; Mi et al., 2014), which cannot be compared with the similar type of scaffolds (cartilage tissue) manufactured by TPP (Figure 7) in terms of precise geometric definition or spatial resolution.

## Conclusion and future perspectives

To conclude, in the past 2 decades, since the close collaborations of physics, chemistry, materials science and mechanical engineering, this multidisciplinary field has made considerable progress. Especially in the past decade, great support was provided for developing TPP qualified as biomedical 3D scaffolds, having well-defined 3D structure with controllable shape, pore dimensions, resolution, mechanical and biochemical properties. However, there are several limitations of the TPP technique that preclude it from the further applications through the biomedical 3D scaffolds. The main limitations include 1) the limited overall size of the structures fabricated by TPP 2) the inadequate throughput of TPP processing 3) the shortage of applicable biomaterials for fabricating biomedical scaffolds by TPP. The whole processing of a mm-scale construct with submicron spatial accuracy might take from a few hours to days, depending on the complexity of the structures and the photoactivity of applied material.

In terms of mechanical engineering and physics, piezo stages are often used as a high-precision motion system to meet the high resolution of TPP processing, however, the scanning speed and accessible field of view using piezo stages are far less than millimeter level. Adding galvanometric scanning mirrors can improve X - and Y-dimensional writing speeds (up to several cm per second) but fail to address the limited accessible field of view. Introducing linear stages can stitch together the multiple fields of view made by scanning mirrors or piezo stage but also introduces stitching errors. Another strategy of synchronous movement of galvanometric scanning mirrors and linear stages can solve the problem of stitching error and improve the writing speed of TPP to a certain extent (several cm per second). High-performance air-bearing stages can greatly improve the scanning range and speed of TPP. However, it only solves the issue of limited overall size, because when the writing speed is increased to more than 10 cms^−1^, inertia will seriously affect the spatial resolution of complex structures and cause defects. This may be the reason why the scanning speed of TPP can be increased to 1000 mms^-1^ with thiol–ene click chemistry mentioned in the previous section, but only simple structures such as cubes and rings were fabricated by TPP. Therefore, improving the throughput of TPP by greatly increasing writing speed is limited by the physical principles. So far, there are many other efforts, such as shell fabrication approach, model replication method, microlens array (MLA) parallel manufacturing and so forth, to improve the overall throughput of TPP (Baldacchini et al., 2021), however, these methods have not completely solved the problem. As 2D light projection has developed laser scanning into stereolithography, the current scanning method of TPP can naturally develop into 3D holographic projection. Therefore, the use of spatial light modulator (SLM) is the most promising solution that can be predicted at present for improving structure size and throughput. The main difficulties faced by this method are the generation of holograms and the high laser power required to generate 3D structures, the solution of these problems will depend on further advances (e.g., improvement of software and algorithms to run the SLM, development of laser technology) in mechanical engineering and physics. Currently, combining TPP with other techniques such as single photon polymerization to produce 3D biomedical scaffolds with larger overall size and more complex local features is a practical method to solve the limitations of TPP.

In terms of chemistry and materials science, applications of synthetic and naturally-derived materials will persist. In addition to a single material, the combination of various materials will essentially be further developed. In particular, the recombinant peptide with structural stability with low risk of introducing immune responses could be further studied. The thiol–ene photochemistry for TPP should also be extended to a wider range of materials and further characterization of structure and resolution. Moreover, although there are currently a number of photopolymers used in biomedical scaffolds for TPP, existing toxicity of the unreacted monomers, oligomers, degradation products and PIs cannot be ignored. These further works require biologists, chemists and mechanical engineers to cooperate more closely across disciplines to meet to meet the requirements for specific physical, biochemical, and geometrical properties required by specific biomedical scaffolds. In addition, as mentioned above, the shortage of highly reactive materials is one of the bottlenecks to improve the writing speed and reduce the energy consumption of TPP. Upscaling of TPP processing efficiency relies on the utilization of highly efficient photochemistry, including the bulk photopolymers as well as PIs. Photopolymers with superior photoreactivity and PIs with high initiation efficiency especially water-soluble PIs need to be further developed for TPP in biomedical field, which will benefit from chemists’ full understanding of the relationship between molecular structure and TPA properties.
